# Expert views about missing AI narratives: is there an AI story crisis?

**DOI:** 10.1007/s00146-022-01548-2

**Published:** 2022-08-25

**Authors:** Jennifer Chubb, Darren Reed, Peter Cowling

**Affiliations:** 1 Department of Theatre, Film and Television, University of York, York, United Kingdom; 2 Department of Sociology, Digital Creativity Labs, University of York, York, United Kingdom; 3 Digital Creativity Labs, Queen Mary University of London, London, United Kingdom

**Keywords:** Narratives, Futures, Artificial intelligence, Storytelling, Technology

## Abstract

Stories are an important indicator of our vision of the future. In the case of artificial intelligence (AI), dominant stories are polarized between notions of threat and myopic solutionism. The central storytellers—big tech, popular media, and authors of science fiction—represent particular demographics and motivations. Many stories, and storytellers, are missing. This paper details the accounts of missing AI narratives by leading scholars from a range of disciplines interested in AI Futures. Participants focused on the gaps between dominant narratives and the untold stories of the capabilities, issues, and everyday realities of the technology. One participant proposed a “story crisis” in which these narratives compete to shape the public discourse on AI. Our findings indicate that dominant narratives distract and mislead public understandings and conceptions of AI. This suggests a need to pay closer attention to missing AI narratives. It is not simply about telling new stories, it is about listening to existing stories and asking what is wanted from AI. We call for realistic, nuanced, and inclusive stories, working with and for diverse voices, which consider (1) story-teller; (2) genre, and (3) communicative purpose. Such stories can then inspire the next generation of thinkers, technologists, and storytellers.

## Introduction

Since the dawn of language, we have made sense of the world through stories, narrating our experiences so as to understand the past, present, and future (Mead 1959). It continues to be true that anything in human experience can be interpreted as a story or narrative (McAdams and Guo 2015). In the case of technology development, stories alter and reframe our expectations. These ‘socio-technical imaginaries’ (Jasanoff and Kim 2015) generate new opportunities by influencing perception and public policy (Hudson, Finn and Wylie, 2021; Dillon and Craig 2021). Fictional narratives influence current decisions that in turn affect the future. Collective stories are central to the human condition (Kearney 2002). They open up new possibilities and perspectives but also divert and distract. This is particularly pronounced as ‘AI narratives’ (Cave et al. 2018), literature, and the arts become “a new source of moral, political and technological imagination” for “technomoral futures” (Vallor 2021, p.1).

Narratives about the potential of AI vary from works of science fiction and corporate marketing by big tech firms to subtler storytelling told by scholars and public intellectuals in popular academic books and documentaries e.g., Noble (2018), Crawford (2021), Benjamin (2019) and O’Neil (2016). The title of this paper references a participant’s views that there is an AI “story crisis” in which stories are being told to further specific agendas, particularly by those in big tech and Silicon Valley. While ‘crisis’ might not be the most accurate description, an exploration of the influence of narratives may help us avoid dramatic or unwanted outcomes. In a recent paper, Hudson et al (2021) described the importance of science fiction in AI policy-making, stating that “stories we tell about AI have foreshadowed and heralded the emergence of these technologies by years, sometimes by decades” (p.1). With Governments all over the world publishing aspirations for AI—including the UK’s recent National AI Strategy 1—it is clear that narratives can play a critical role, in helping us separate hype from reality (Milne 2020). For this reason, narrative responsibility, or what we might refer to as responsible storytelling, is an emerging field of AI ethics (Coeckelbergh 2021). Though out of scope of this paper, there are questions to be asked about the responsibility of science fiction authors to consider their influence on public awareness of technology (Fast and Horvitz 2017).

Two prominent voices in AI narratives research are Stephen Cave and Kanta Dihal (Cave et al. 2020). They suggest that socio-technical imaginaries (Jasanoff and Kim 2015) should include an explicit account of the important role that narratives play and assert the need for further investigation into the ways narratives impact upon the public. This is supported by a growing body of research into the critical value of AI narratives (Kim 2022; Coeckelbergh 2021). We add to this critical line and Cave et al.’s work by focusing on novel, nuanced and niche AI narratives.

This paper offers original research on academic perceptions of the role of AI narratives in contemporary culture. We present the results of interviews conducted with leading scholars interested in AI futures. We argue that what is missing is the kinds of AI narratives that would both engage with the lived realities of these emerging technologies and also help advance more positive and just implementations.

We contribute to the literature by detailing empirical data from interviews (*n* = 25), in which leading scholars from a range of domains including AI and Computing, Science and Technology Studies, Interactive Media, Literature, Education and AI Ethics describe common or dominant narratives, perceived by them to influence the public discourse on AI, before exploring missing or alternative narratives under the rubric of novel, nuanced and niche. To do this, we first discuss current AI narrative research. We then introduce our conceptual framework and empirical methods and present our findings about missing, nuanced narratives. As ‘dominant narratives’ are known, polarised, and established, participants use them as a foundation on which to build the future direction of AI discourse and development. We find that what is missing are narratives and stories about what people might *want or hop*e for from AI in their *everyday* life; these niche stories are often suppressed because they are less sensational, and hence less valuable in capturing attention and advertising revenue. We argue that central to the promotion of missing narratives is a question about *who* ought to feature in them and who is telling them and why, e.g., the way power operates dictates the way stories develop and inspire. It seems critical to consider who controls the dominant narratives, and who stands to gain from these narratives (this may often be the same person — consider Zuckerberg or Musk). There is then the need to make it harder for those who benefit from the narratives to influence what the dominant narratives are (Crawford 2021). As such, narrative features, such as (1) the storyteller (2) genre and (3) communicative purpose, should be explored. Efforts to produce new and responsible stories about the future of AI should continue to be made which challenge the dominant tropes outlined here. Through this work, we identify and justify stories as identified by scholars in related fields of AI and AI Futures and call for further research to inspire and benefit society.

### Previous research on AI narratives

There is a long narrative history of AI and a number of works focusing on portrayals of AI or intelligent machines (e.g. Kang 2011; LaGrandeur 2013; Devlin 2018; Hermann 2020; Truitt 2015; Ward 2018). There has, for some time, been a clear focus on public perceptions of AI influenced by broader narratives (Cave et al. 2018; Fast and Horvitz, 2017; Kelley et al. 2021; Zhang and Dafoe 2020; Cave et al. 2019). Not least, AI in fiction has been collectively consumed by the public, across generations and cultures. Although the existing literature on non-anglophone imaginaries of AI is sparse, there is growing interest within academia and beyond because of the impact they can have on sense-making (Felt 2017). The philosopher Stephen Cave, for instance, writes about how narratives can become “entangled” with real-world developments. In the case of AI, the study of narratives is “entangled with the emergence of AI and robotics” particularly in the Anglophone West (Cave, Dihal and Dillon, 2020). The role of science fiction in futures research is regularly criticised as misleading. Utopian/dystopian thinking, while often desired for its attention-grabbing properties (Jameson 2005), is associated with notions of fantasy and embodied Superintelligence and concerns about its oversimplification (Cave et al. 2020, p.6). Cave et al. describe how “narratives of intelligent machines matter because they form the backdrop against which AI systems are being developed” (p.7). Citing a 2008 AI Report to the Select Committee, they assert that current narratives are often “out of kilter with the present state of the technology” (ibid.). Indeed, these narratives and stories can pervade public perception (Cave et al. 2018; Cave and Dihal 2019). A study by the Royal Society suggests there is an urgency to take AI narratives seriously to improve public reasoning and narrative evidence (Dillon and Craig 2022). What is required, they suggest, is ‘story listening’—an active engagement with, and anticipation of, narratives as a form of public participation, where imaginaries of AI facilitate public reasoning and inform policy. Narrative plays an integral part of these visions, and they are shifting all the time (Bory 2019). Of course, what stories and narratives have in common is their tendency to be the subject of hype (Blom and Hansen 2015; Samuel et al. 2021; Slota et al. 2020). Hence, there is not only a requirement for new narratives, but also an increase in public understanding about the need to interrogate narrative features: who is telling the story, what is its genre, and what are their communicative purposes?

Research on AI narratives has revealed concerns about unintended consequences and injustices. For example, as gendered AIs populate popular culture (Yee 2017; Cave et al. 2020; Devlin 2018) and our homes (Alexa, Cortana etc.), questions are rightly focused on who is telling the stories that are informing our sense-making. Indeed, the extent to which these fictional narratives inform and engage with issues of gender and race (Cave and Dihal 2020; Cave et al. 2019), and ascribe a particular view of race and ‘whiteness’ (Katz 2020), is of ongoing concern and debate in wider attempts to decolonise AI (Noble 2018; Benjamin 2019).

The mainstream media continue to reinforce public narratives about AI as ‘scary robots’ (Cave et al. 2019, 2018) with an increasing but ‘shallow’ focus on ethical implications and issues of representation (Ouchchy, Coin and Dublijevic 2020). Portrayals of AI in visual and sound media are often dystopian. The sonic framing of AI, presented via eerie music in film, reinforces a view of an AI ‘uprising’ or some form of subtle manipulation by AI agents. The sonic framing of AI, then, combines with and reinforces stories of malevolence and danger. (Forthcoming, Chubb and Maloney 2021).

In this paper, we build on Cave et al’s (2020) work on AI Narratives by identifying stories which ‘contrast to the narratives that currently dominate’ (p.6). We do so by focusing on missing narratives identified by leading scholars in the field of AI. These narratives become the spur to think about strategies for story development, embracing the notion of human flourishing and abundance, where these relate to the intimacies and contextures of people’s lives.

### A conceptual framework for AI narratives

To reinforce the work of Cave et al (2020), we build on a framework, established by Sheila Jasanoff, that pursues the view that ‘sociotechnical imaginaries’, or shared visions of futures related to science and technology, embed science and technology in social processes. These imaginaries are “collectively held, institutionally stabilized and publicly performed visions of desirable futures, animated by shared understandings of forms of social life and order attainable through and supportive of advances in science and technology” (Jasanoff and Kim 2015, p.4). Such visions of “desirable futures” are often those promoted by governments which become highly prevalent in everyday life.

Jasanoff’s imaginaries “encompass both positive and negative imaginings” (Jasanoff and Kim, 2015, p.3). Cave, Diahl and Dillon (2020) asserts that with AI narratives these only act “in service of the dominant vision” …. (p.6). This might be that of science fiction (Robbins 2016; Mayor 2018; Kress 2001). For instance, sociotechnical imaginaries are often attached to imaginaries beneficial to human needs (Wade 2018) and allow for the development of understanding of problematic aspects in relationships between science technology and governments. Through this lens, we can critically challenge existing sociotechnical imaginaries to consider what values should be promoted through technologies. Rather than taking for granted the dominant line of desirable or undesirable visions—in the case of AI narratives—we can consider which visions are missing or diminished, and what values stories ought to promote. Using data from our interviews, we illustrate a broader set of future visions from experts.

### Selection, recruitment, data collection and analysis

This paper reports on the findings from (*n* = 25) online interviews with academic leaders and scholars in AI and AI futures and related domains including literature, media and engineering. A full list of these disciplines can be found in previously published work, Table 1. (Chubb et al. 2021. p.4.). Interviews were organised following a comprehensive review of the literature on AI futures, AI and impact, and AI and society, and a mapping of the relevant research institutes, centres, and universities. Interviews were conducted online following an adjustment to the team’s research methodology in light of the COVID-19 pandemic. Ethical approval for the project was sought and granted and participants consented to anonymous participation.

**Table 1:** Mentions associated with thematic coding of alternative narratives

Themes	Mentions associated with themes
Culture, art, and creativity	Games, AI in music, eXtended Reality (XR) and interactive storytelling, dance AI to represent art and music, AI as a musical tutor, AI written novels and avatars
Science and education	AI in science, AI and robots in space, information retrieval, interdisciplinarity, public intellectualism and big data
Practical and everyday	Mundane tasks, gardening, cooking, cleaning, repetition, work, individual day to day life, dangerous work, tidying, heating, watering lawn and logistics
Relationships and community	Dating, friends, match-making, networks, voices and community
Environment	Climate change, species extinction, global risk, ecology, sustainability, climate models, bird migration, conservation, greenhouse gas emissions, digital footprint, food stability and veganism
Health	Care for the elderly, applications in psychology, COVID-19, robots in care, Fitbit and timers to change posture
Social justice	Community, equality, dialogue, fairness, unfairness, gaps, systemic bias, social capacity and capability, complexity, oppression, risk, racism, privilege, whiteness, male, gender, control, diversity, education, liberal, unconscious, uncertainty, race, colonialism, representation, discrimination, design, historical bias, implicit bias, feminism and Black Lives Matter
Spirituality	Self-monitoring, meditation, zen, time, green spaces, Buddhism, enlightenment, thriving, happiness and ‘filtering out’ the world
Economics and policy	Tensions, solution, growth, responsible innovation, solving intractable political problems, austerity, Universal Basic Income, public services, NHS and wellbeing

Participants represented a spectrum of disciplinary backgrounds across the social sciences, humanities, natural and life sciences and engineering, physical sciences and mathematics. We sought balanced participation in terms of gender and race. Despite the preponderance of the male gender in some disciplines (Stathoulopoulos and Matteos-Garcia 2019), sixteen out of twenty-five (64%) of our participants did not identify as male. Criteria for inclusion included proven expertise within AI through academic publication and current position within a research organisation or HEI. Participants were emailed with a schedule, an information sheet, and informed consent form. No one refused the study directly though several did not reply.

### Limitations

This paper is not a study of the actual narratives present in the world, but an analysis of what a community of those professionally engaged in researching AI and ‘AI futures’ believed were the dominant and overlooked narratives. This sample could be seen as a limitation in design, with participants speaking as insiders on a topic. We aimed to energize public narratives by speaking to people who understand those views because they navigate them all the time. For this reason, the participants were well placed to provide a view as they typically act as mediators, a link between what people think about AI and the reality in particular domains. 

A second limitation is in the acceptance of varying definitions of ‘AI’ and ‘narratives.’ This paper discusses AI futures across a range of domains in which there are different uses of those terms. Future research could consider specific definitions and associated needs in particular domains when creating new narratives.

### Analysis

This paper reports on the deductive findings from the interviews in which participants were asked to describe: (1) the commonly told stories or dominant narratives in AI? (2) the extent to which these may or may not differ from reality; (3) who features in the narratives?; and (4) who is telling stories about AI? We also asked which narratives were missing or diminished. Appendix 1, provides a complete interview schedule with questions and themes for a semi-structured style interview conducted online in the summer of 2020.

Deductive and inductively coded thematic analysis (Braun and Clarke 2006) was combined with qualitative data analysis (Miles, Huberman and Saldana 2014). Appendix two provides an overview of the ‘parent ‘coding for the entire project and the nodes on AI narratives. For the purposes of attributing participant involvement to verbatim quotation, we provide disciplinary field information and a unique numeric indicator. All interview data were anonymised at the time of analysis, with individual identifiers used to denote verbatim quotations. Consent was gained for the audio-recording and transcription of interviews. Data were stored securely on a password protected computer with recordings deleted after use.

## Findings

Our research supports the view that AI narratives and stories play an important role in conceiving of social order, but at present, scholars feel that dominant narratives polarize toward notions of threat or a kind of myopic solutionism. This was regularly reflected in the accounts of experts, where there were repeated references to words like ‘paradox’, ‘polarization’ and to extremes:

*The narrative that says this is the sort of silver bullet if you like is going to solve these intractable political problems or embodied robots taking our jobs. [Social Science 10]*

On the one hand, stories are seen to portray AI as a silver bullet, on the other, they are the very origin of moral panic. Experts suggest that most positive stories about AI are either (1)* overly optimistic,* or (2) *attribute magical qualities*. With the first, AI is often positioned as the answer to all the world's problems seen in its application to grand challenges (Anderson et al., 2018). With the second, AI is a form of voodoo, rooted in fiction, appealing to our imagination and fantasies. On the one hand, this might help us make sense of what we want and on the other distract us from clarifying the role AI could take; one person’s utopia could well be someone else’s dystopia (Cave et al. 2019).

Experts suggest that negative stories about AI are either (3) *existentially spectacular*, where AI will take over and surpass human intelligence and become super-intelligent, or (4) *individually threatening,* where AI is portrayed and understood as a risk to our livelihoods and safety. Positioned at either ends of a spectrum, these positive and negative narratives leave an intervening space that might be filled with more nuanced and novel stories. In turn, these alternative, and currently missing, narratives can provide a more realistic means to understand and anticipate a beneficial and equitably future.

Participants unanimously felt that such polarisation was unhelpful and in almost every case the result of hyped-up storytelling by the media, science fiction writers, or big tech companies who want to portray a particular view of a ‘good or bad use’ of technology, to attract (monetized) attention (Samuel et al. 2021). Instead, experts felt stories about AI that were more responsible and nuanced were required for improved understanding of AI:

*I think narratives polarise and I think that’s unhelpful because for me the debate needs to happen in a way that allows us to acknowledge that in almost every instance that we’re going to be using these sorts of methods and technologies, there will be things that we conventionally think of as ethically good or bad, those things will be entangled still. [Social Science 10]*

The kind of entanglement described is well articulated by one participant who claimed that we need to view the technology in less binary terms noting that technology is neither intrinsically good nor bad:

*I think that what we need to be able to do in this space is to keep in our minds those two things simultaneously that actually the same technology that is detecting some movement in the terrain from a video feed of a drone is actually being used in the video feed linked to the CT scans and the MRI scans in hospitals. [Social Science 10]*

Instead, a more nuanced approach to stories could be taken, one which ascribes less rhetoric about what is good or bad but one which is interrogated for bias and communicative purpose.

*I suppose one thing that comes across is avoiding making categorical claims about the good or sort of being attracted to kind of headlines around the good and being willing to kind of be more nuanced about what we say about AI. [Social Science 14]*

When asked about ‘dominant narratives’, these were treated by the respondents as known, polarised, and established. Each of the participants worked from this position (in part because of the interview schedule) and looked to say something deeper about AI Futures. Nuanced and novel stories are missing, they claim. We first explore what those dominant narratives are as described by participants before discussing missing narratives.

### Dominant narratives

Analysis reveals antipathy towards ‘commonly told’ AI narratives, this includes a tendency towards a dismantling of 'grand narratives' of AI and the very term AI as a placeholder. In particular, participants expressed that dominant views of AI are polarised as a result of the portrayals propagated by the narratives we read, watch and consume via popular culture. Such views distract the public from understanding the current capabilities of the technology, which, while entertaining, may also be disproportionate and disruptive. For instance, superintelligence and existential risk narratives while important, may distract from the pressing issues of the day:

*You can talk a lot about different aspects of how an AGI might emerge and how this might cause negative outcomes and you can paint some very vivid pictures with thought experiments and... I don’t know if you’ve read Max Tegmark’s book or Nick Bostrom’s book but they have wonderful stories within that. So that makes it a very easy thing to talk about but just because it’s simple and easy doesn’t necessarily mean that that’s actually where the greatest risk lies. [Arts and Humanities 15]*

Dominant narratives were for most, based not on the reality of the state of the technology, but on the speculative imagination of science fiction authors:

*Well, certainly I would say in Hollywood and science fiction the two main narratives are basically the Pinocchio story which is like Commander Data in Star Trek Next Generation, the mechanical thing that wants to become a real boy, wants to have human emotion, wants to become human and then there’s The Terminator scenario or somehow the AI uprising scenario. Both of those are much more about people than they are about AI, they’re not really rooted in reality because I think of artificial intelligence primarily as a set of tools for building software so in that sense it’s a different kind of software engineering and I think whenever you’re thinking about an AI system and a story, if you replace the word AI with the word software does it still make sense? [Science 03]*

**Fig. 1:**
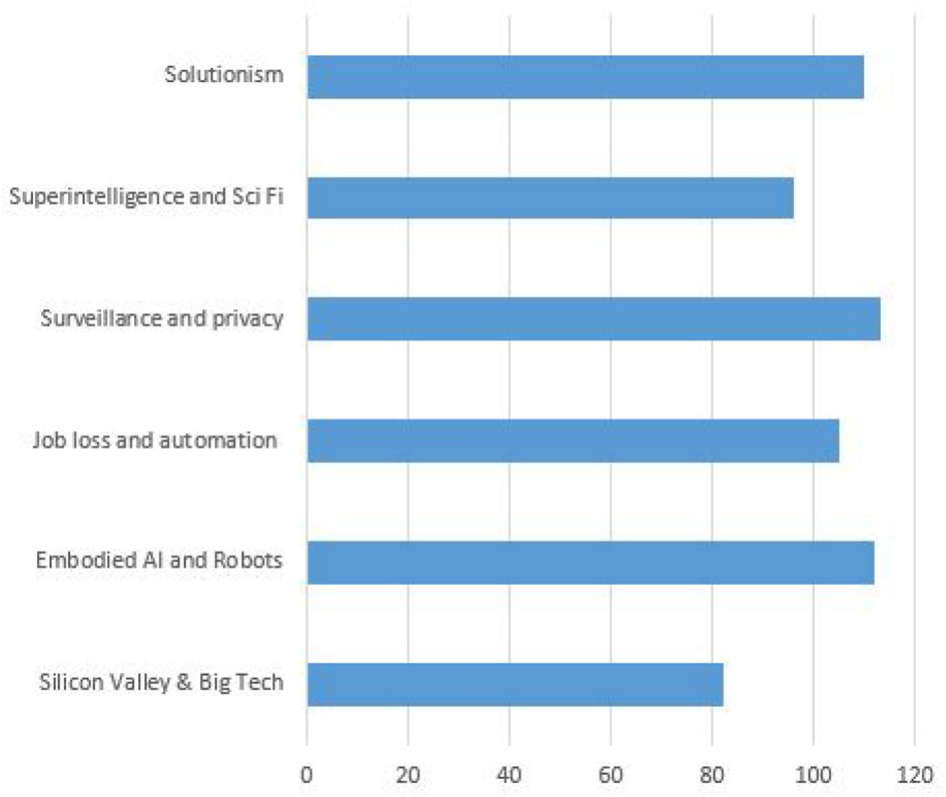
Dominant narratives by content analysis

Figure 1 shows the number of references made by participants when asked about common public narratives. Participants referred to superintelligence and sci fi, embodied AI and robots and the prevalence of Silicon Valley and big tech. This is perhaps not surprising with the rise of technologies, such as facial recognition and social media networks. Less commonly referred to narratives included clickbait, job losses, efficiency boosts and the use of the words dystopia and utopia. Largely, the common narratives were seen to fall into the former category (dystopia) with the exception of solutionism, which was seen as a bright-siding alternative where AI would simply solve all humanity’s problems. The remaining categories are now explored further.

The following section describes some of the ways in which participants described these dominant narratives about AI. The findings provide a backdrop context from which participants navigate toward new narratives. The dominant narratives frame the other findings that follow. Future research might test out the prevalence or accuracy of the dominant narratives as perceived by a broader set of experts.

### Surveillance and privacy

First to emerge from the interviews was the extent to which participants felt that narratives about surveillance and privacy would be at the forefront of people’s minds when thinking about AI:

*I think the narrative of either the inventor in the garage narrative, or I think much more common now, the couple at multinationals who are using data, violating privacy, kind of generating surveillance capitalism and so on, all of that kind of rise of corporatism and the loss of the individual and the weakening of the state, dovetailed to the building of this very powerful technologies. [Social Science 10]*

Several mentioned stories relating to the manipulation of data, such as those depicted on screen in the *Big Hack* and the *Social Network Dilemma,* describing the Facebook and Cambridge Analytica scandal and asking “*How can we all be anonymous and safe?” [Social Science 17].*

Most felt that the benefits of AI came at a cost. This was closely linked to concerns about loss of power, freedom and manipulation:

*We obviously are seeing some of those stories, I think mostly about AI in policing and surveillance but also obviously AI in advertising and, you know, optimising the wrong thing. [Arts and Humanities 03]*

In particular, everyday privacy was a concern when considering the effects on vulnerable or marginalised groups in society:

*What do we want from AI? The clearer question is who do we include in that ‘we’. Does it impact upon some groups more than others? [Arts and Humanities 04]*

Experts felt they had a role in ensuring that those inequalities were exposed and subverted. Stories about AI, ought to serve a broader social purpose to tackle these concerns.

*For me, every use of AI is a subversive one, right, which is, and has to be one about fixing a broken system, so to speak, or replacing a broken system with something else. Narratives need to support that. [Arts and Humanities 09]*

In a sense, these findings reflect their roles as experts as less is known about how the public relate concerns about privacy with AI. There were also comments that AI was subject to so much hype that it was often presented as the solution to all problems.

### Embodied AI and robots

This theme associated AI narratives with embodied, anthropomorphised robots. Associations made to Frankenstein’s monster, non-human, cyborgs were coupled with predominantly negative connotations, such as ‘disturbing’, ‘misleading’, and ‘killing’, were made more readily than connections to robots as ‘companions’, ‘carers’, and ‘affective systems’ unless they directly referred to healthcare robots:

*Highly anthropomorphised machine in a robot body that is humanoid either as a servant or as a warrior killer robot kind of Terminator type story but these are the things that kind of serve us or live with us or trying to exterminate us but they are very much, kind of embodied, I think that’s a very common and very problematic narrative. [Arts and Humanities 12]*

Again, and unsurprisingly, science fiction was mentioned as most influential in shaping public perception. The most commonly referred to example was The Terminator, followed by HAL in 2001: A Space Odyssey, and then more general references to ‘Hollywood’ and films like Ex-Machina, Alien, Wall-E and i-Robot.

*You know, this is sci-fi-inspired fear, so, I mean, perhaps, to some extent, legitimate about robots taking over. The Terminator scenario or somehow the AI uprising scenario. [Arts and Humanities 13]*

What characterised these narratives, participants felt, were mentions of evil AI, invisible programmers, or aliens:” *AI is sort of an alien type of being that becomes, you know, sometimes evil” [Social Science 02]. “*Man versus machines” narratives were also dominant; often participants suggested that such stories tended to depict programmers making errors in the design of systems leading to bad outcomes, e.g. 2001: A Space Odyssey.

*The system put the outcome of the mission as more important than the lives of the crew, the human crew, and that’s an error of specification from a software standpoint. [Science 17]*

Further, several commented that what was common across these science fiction narratives was that designers, programmers, and even the operators are often invisible. This heightens the human versus the machine narrative and maintains the narrative of moral responsibility:

I* mean people refer to bureaucracies as machines themselves and that’s of great concern obviously from the point of view of maintaining moral responsibility for the actions of the system rather than blaming everything on, well, we can’t do it because our computer system won’t let us. [Science 12]*

Additionally, stories about embodied robots were generally seen as a cause for concern because of the racialised and gendered stereotypes. Films tend to over-sexualise female androids (e.g. see Ex Machina and the male-dominated world Ava (the robot) is seen to threaten), or project a view of AI as white, suggestive of a lack of representation.

*A lot of cis white men write science fiction, and that perspective tends to get engrained in the science fiction stories that are told, and so we get science fiction AI stories dealing specifically with these kind of fears, and I have seen it. [Science 06]*

Concerns about portrayals of robots in science fiction extended to a further consideration that the demographics of storytellers in AI as being largely elite, white and male — this is particularly the case in Silicon Valley and Big Tech. As such, participants regularly couple concerns about narratives issues of bias both in terms of stories told and the algorithms themselves.

## Job loss and automation

Some participants referenced how the public likely associates AI with negative implications, ranging from job loss (e.g. from the rise of autonomous vehicles and more generalised automation) to what a few referred to as human ‘enfeeblement’, e.g. where humans would become subservient and powerless to AI. Positive associations about increased efficiency for improved freedom, and solutionist narratives where AI is the answer to climate change and the COVID-19 global health crisis were also among more positive framings while job loss too also posed a potential positive future:

*So, we are not cleaning the streets, we are not doing the garbage, we are not doing taxes, we are not selling things to people – all that stuff is AI. And, we are sitting around painting and inventing new religions and looking pretty! [Media, 18]*

What was also common was a tendency for our participants to hint at alternative narratives in their responses. These were either relative and nuanced, or missing altogether.

### Superintelligence and science fiction

Another theme when asked about dominant narratives was that the public would probably see AI in relation to a kind of ‘uprising’ of robots associated with speculation in science fiction.

*So I do think those words “artificial intelligence” summon up the kind of Terminator and that kind of robotic humanoid robots. [Arts and Humanities 22]*

Most felt these were not likely scenarios and that AI was “narrow” and at a lower-level, far from anything like superintelligence – a view where AI would exceed human intelligence:

*I’m talking about things like robot vacuum cleaners being anthropomorphised, attributed to agencies, being seen as the first step towards superintelligence. [Arts and Humanities 04]*

Narratives about such scenarios were seen as desirable and led to many participants describing concerns about human-AI value alignment. Several felt that current dominant narratives reinforce imaginaries of a human versus machine construct:

*I almost find it very difficult to imagine a world, you know, where some, you know, super-intelligence would cohabitate peacefully and well with humans, especially one that’s created by humans to serve their best interest at first. I think that would be very complicated. I’m not very optimistic about that. [Social Science 02]*

When asked about superintelligence, almost all participants described how such notions were more suited to the realms of fiction than reality:

*I think the question of human-level AI and human-plus-level AI is really more of a question for science fiction than anything we can realistically expect to reach. [Science 06]*

A more realistic narrative of AI futures, one claimed, would be more true to what’s actually happening in Silicon Valley:

*It’s going to be more like Google than like The Terminator. It would be really hard and really pointless to try and turn everything into embodied robots. [Social Science 15]*

## Silicon Valley and ‘Big Tech’

It was felt that what is happening in Silicon Valley and other Big Tech companies reflects some of the grander (and scarier) narratives found in science fiction:

*I think all the stories in this space that’s dominated by Silicon Valley. The big problem is the tech industry in my view. The Olympians of AI. [Social Science 23]*

The companies most commonly referred to were Amazon, Facebook, Google, Apple, Facebook and Microsoft—all seen to be the loudest voices and the most influential of the storytellers, whose communicative purpose to sell and market technology was seen only to manipulate and be driven by the profit motive:

*It’s dumb AI but in the hands of powerful people whose motivations are highly questionable because it’s basically me and myself making shitloads of money, it’s extremely problematic. [Science 08]*

The most common theme when discussing big tech inevitably relates to accountability and responsibility. Few participants said anything positive about such companies. With respect to stories, participants felt that such companies over-sold the capability of AI and that the profit motive was harmful to humanity:

*it really comes down to the role of technology in the economy. Are we selling technology to make money for ourselves and our bosses? If we are co-existing in a free market-ish economy, that is going to be heavily driven by taking as much from us as possible, for as little value as possible – that is the goal! [Arts and Humanities 16]*

The personalities involved in such companies e.g. Zuckerberg, Bezos, Musk, etc. were seen as incredibly powerful—typically white, privileged and male, representing a narrow social group with, largely, shared ideologies:

*Elon Musk has got such a huge number of fans when he’s clearly a not that nice human being: Abolish Silicon Valley! [Science 08]*

## Narratives are relative

We find that all narratives are relative to audiences of different ages, geographical locations, and abilities. They are also relative to the domain in which they operate and the purpose they have. One participant talked about how the cinematic portrayal of AI has shaped generations of people’s perceptions of AI and mused about how dystopian science fiction of the 80 s/90 s, might have a different effect to the socially minded robot Wall-E cleaning up the mess humanity had created.

*What if our earliest AI stories had not been 2001 with Hal and Terminator, with Skynet, or if had had WALL-E as our first AI story, how different would that have been? Or, some of the Japanese AI characters who are generally not murderous? [Science 06]*

The participant noted that this is not just generational, but is also cultural, describing how AI is seen more as a friend to those in Japan, for instance, compared to the West:

*You might think that everyone in the world feels like this about AI and robots, but this isn’t true. So, it is very much a Western European and descendants type preoccupation which goes back to some of the myths of Greek society, if you look at the ancient Greek Talos, which is a big metal challenging thing, has popped up all the way through in stories, all the way through the period because the technology is now better, the stories made seem superficially more plausible. ... People in Japan don’t feel like this incidentally, so this is just another version of the Frankenstein complex. [Arts and Humanities 04]*

Responses to dominant narratives are shaped by personal experience, culture and age, for instance. Our participants stressed how AI narratives ought to speak to, and be shaped by, a range of individuals of different race, cultural background and ability:

*I think the problem is not so much people asking do we want this as making them wonder about who is included in that ‘we’ because I think with most of the things that are being developed the question do I want this is answered with a resounding yes, the stakeholders in self-driving cars want self-driving cars, the problem is asking those who don’t have a say in the development or implementation those who will experience a radical change in their lives when these technologies become implemented, those are not included in the ‘we’ and that is where the question of do others actually also want this or is it just the five of us in a rented office in Silicon Valley? [Arts and Humanities 04]*

For instance, for one participant who described a disability, the idea of a self-driving car was transformative. For them, stories about freedom through the advent of autonomous vehicles were appealing, whereas their partner, might have a different view:

*I’m waiting for the day for autonomous vehicles because I have very, very poor eyesight, I’m never going to be able to drive, and that has had a big impact on me. I have to say my partner hates the idea of autonomous vehicles, they’re very distrustful of computers and... I could never imagine them feeling comfortable in an autonomous vehicle, whereas I imagine I would really want to get one as soon as they’re available. [Arts and Humanities 18]*

For participants, these binary positions were adopted by the public in response to notions of embodied, super intelligent AI depicted in science fiction and cinematic stories in everyday culture. Interviewees spoke in detail about the need for more nuanced stories that challenge those pervasive narratives. Missing are narratives and stories about what people *hope for* and *want* from AI. So too are questions about *who* should feature in these stories. We envisage a domain in which niche stories—so often under-reported because they are less fantastical—flourish and grow; one that advocates for the inclusion of people not normally associated with AI stories.

## Missing narratives?

Our participants felt that there is a need to focus on and gather niche, novel and nuanced stories and narratives about the impact of AI, grounded less in the spectacular and more in the everyday.

While the experts disagreed about the relative likelihood of a super intelligence (Statton and Milford 2017), they agreed that the topic drew attention away from the opportunities of AI and the inclusion of critical and diverse voices. To them, narratives should focus on the realities of AI’s present capabilities rather than unlikely, threatening or exciting futures. These narratives can then lead to greater public involvement through education and information. Stories about ‘narrow AI,’ as opposed to fantasies of superintelligence, were seen as an important means to ground people in present realities and issues. This is seen in an exchange between one participant and the researcher.

*Participant: AI is presented as a threat, both in the short-term for jobs and in the existential*

*‘Humans are doomed!’ type setting. Both of these threads are very annoying if*

*you work in the field.*

*Researcher: Can you tell me a little bit why they annoy you?*

*Participant: Because they are completely untrue, broadly. None of these systems can do anything like what most humans can do. In particular, they are not flexible, they don’t have wide scope, they can do specific tasks narrowly defined better than humans. substitute for humans – this is just nonsense! Absolute nonsense! [Science 07]*

Our participants described areas where AI could benefit society and the areas where there were missing narratives. What if, one participant exclaimed, the first story you were told about AI was one of human flourishing instead of human demise or replacement? To what extent would that shape how we view the role of technology in our lives. Importantly, all interviewees were reflective of the fact that AI stories have a long narrative history and that this influences public perception in significant ways. As one participant stated, “*amazing things happen when enough people believe in a good positive story.” [Social Science 02].*

### Co-produced collective, responsible and creative storytelling

There was recognition that such stories would not suffice, instead stories should be co-created—perhaps based on prediction, using forecasting tools e.g., Metaculus,^2^ to explore human predictions and goals:

*I think that’s fascinating, to imagine, you know, young people sort of really rigorously thinking through those sorts of pathways to different futures. [Social Science 23]*

Participants unanimously voiced the view that narrative and story was important in both understanding and ‘de-bunking’ AI and in working towards futures that enable human flourishing. Many felt it would be ground-breaking to embed teaching of forecasting to train people to think more rigorously about the future. Additionally, the use of alternative forms of expression to tell a story, was deemed a creative and powerful way of engaging people about the realities of AI:

*The artist who worked with us - that it was a very powerful narrative that can engage people to show perhaps what is involved in using, in developing, designing, and using an AI system. How can you say, “Well, this will definitely be beneficial,” when we have a government and we have the planetary conditions that we have, and I am not being a luddite, and I am not being negative, I am trying to be completely accepting of the situation we are in, and it is extraordinary to me that we are not using these tools to ameliorate that situation. That is what we should be doing. [Science 21]*

Many participants called for storytellers and publics alike to look at the current situation with AI and ask how we can use of AI to enhance human flourishing (Vallor 2016). Narratives that probe and look to nuance the answers to these questions can help us get closer to those answers, they suggest.

### Alternative narratives

The starting point for any discussion of alternative narratives is the paucity of those that already exist, or at least those that predominate. The following comment by one participant captures this sentiment:

*It feels like we’re going through a little bit of a ‘story crisis’ in the world these days. [Social Science 02]*

This ‘crisis’, claimed the participant is born of a lack of investment in novel, nuanced, and niche narratives. Indeed, the title of our paper references one of our participant’s views that there is as a bit of an AI “story crisis” where stories are being told to further particular agendas, particularly by those in big tech and Silicon Valley. A term used to imply some level of impermanence, perhaps a ‘crisis’ might not be the most accurate description for what appears to be chronic and stable when it comes to AI narratives? But an exploration of the influence of narratives, they felt, may help avoid dramatic or unwanted outcomes from such a ‘crisis’.

In what follows, we draw on the participants' own attempts to encourage and enthuse through the identification of possible lines of story development.

Participants felt that missing from the AI discourse were stories about mobilising AI for virtuous reasons—for culture, for society and for justice—what we might refer to as stories of abundance. Abundance is intimately tied to people’s daily lives and experiences. Participants noted that while stories of abundance do exist, they are rarely nuanced and contextually tied to people’s lives.

There is, then, a lack of narratives that promote discussion of our collective future in a realistic way. For example, one could imagine a world where AI and autonomous systems reduce the number of hours a person works and increase the time they have for creativity, connection with other people, and leisure. These are contingent and incremental benefits, what one participant called ‘contemporary’ benefits:

*I think one thing that has not succeeded in being very prominent is contemporary benefits of AI partly because they are usually very marginal, right, they make life a little bit easier around the corner in all sorts of different ways and many of them are not applied in the home. We would like to see them in the home, I think people like Google and Amazon and so on would very much like to get into the home and get those services there. But really they’re doing a lot of good work in factories and increasingly in farms and in supply chain management and in postal processing and these are not places where either storytellers or lay people are busy very often so there is a general lack of awareness about what happens in terms of how everything gets into our home and how civilisation functions and most of what AI does it does it in those spaces. [Arts and Humanities 14]*

This notion of mundane or contingent benefit is rarely celebrated because, perhaps, it doesn't make for exciting and emotional storytelling.

*I mean in some sense they are boring stories but in another sense it’s the way AI gets woven into how our civilisation is run—these are just completely missing narratives. [Arts and Humanities 12]*

By its very nature, this ‘interweaving’ is subtle and unnoticed, yet it is precisely stories of this ‘future normal’ that are most needed. For one participant, we should embrace this ‘boring’ understanding:

*A lot of the stories about AI are going to be boring stories. [Arts and Humanities 15]*

Yet the ‘everyday’ does not have to be boring *as a story*. If narratives are to be more nuanced and focused on the everyday are what we need, we need to consider why we do not have them. In a sense, what our participants were suggesting was that perhaps those telling the stories do so without the best intentions. The communicative purpose of the storytelling at this point becomes vital, e.g., the communicative purpose of entertaining in science fiction, for instance, and educating trends toward sensationalism in the media run somewhat contrary to the everyday. There is perhaps also less recognition of a social justice motivation for collecting these stories, though we are getting close to it see, Nobel, 2016; Crawford 2021 etc. Our participants felt that stories needed to move beyond the hype. This is again driven by the storyteller.

*Well I think the positive stories over-emphasise the positives and, depending who I’m talking to (laughs), you know, I would sometimes say that the negative stories over-emphasise the negative. [Social Science14].*

Alternative narratives are the sum of the storyteller, genre and the communicate agenda or purpose – participants urged for the public to consider each component of the stories they are told about AI.

## Opportunities for AI narratives

*I think it’s about using AI to do all the types of storytelling that is not mainstream. [Arts and Humanities 18]*

Broadly participants expressed a preference for narratives that supported the idea of AI as augmenting, rather than replacing human intelligence, and was more closely aligned with moral behaviour, e.g., Narratives supporting truth, justice and fairness.

*I mean, there are a lot of examples, like in medicine or in the justice system etc., but stick to the objective parts and don’t try to make emotional machines or something. [Social Science 11]*

Participants urged that AI narratives ought to tell the tales of more realistic and accurate truths, relating to issues of social injustice and inequalities to shift perception and create positive change. This is in line with Coeckelbergh’s (2021) theorisation of responsible narratives.

*I don’t think that I am the one who stands most to benefit because my life is already fairly privileged and convenient and I think a lot of the potential for AI is actually in making services and functions that, you know, we can pay people to do in affluent societies and extend those to places where that is just not possible at the moment. [Science 12]*

**Fig. 2:**
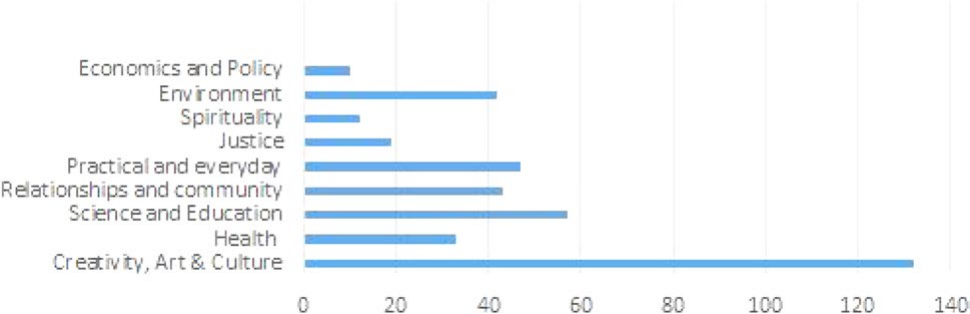
Alternative areas for AI narratives by theme and mention

Against this backdrop, the following themes emerged as important for narrative development. Figure 2 details content analysis with respect to positive or missing narratives as provided by participants.

Figure 2 shows several areas where participants felt stories could focus. Table 2 then shows more detail on the kinds of associations made with each theme during analysis.

Looking at the most dominant areas for new narratives, over half referred to the benefits AI brought to *art* and *creativity*. Though present in current narratives, almost all participants felt that it was there that AI really could be beneficial and transformative. For instance, many described the use of AI as a tool for leveraging human creativity in art, fiction or music.

*It changes what we can achieve, and it changes the music we will write. [Arts and Humanities 13]*

AI was broadly seen as a route to creativity with over half of the participants describing the need for more narratives about AI and creativity in their interviews.

*I think there’s a certain mystery surrounding the act of creativity, but, you know, I, kind of, was able, as a human alone, acting alone without any AI, shall we say, was able to reach, you know, good endpoints, good songs, and we can talk about, you know, what it means to write something that’s ‘good’. But, yeah, I thought, “Oh, I can, kind of, try to, you know, reflect on and implement, in code, some of what I’m doing here. [Science 13]*

Using AI was seen to be fun and interesting, leading people in new directions. AI was seen as crucial to the creative industries where most of the participants felt AI was most beneficial e.g. digital technology, art and storytelling. However, many felt this creativity would permeate to other domains, such as science and education.

A second area of opportunity for new narratives was science and education. Participants saw a role for AI in science, particularly with respect to university research and teaching: “we should be talking about the future of universities” [Social Science 11]. Such benefits in science and education should not be at the expense of human judgment. With respect to teaching and learning, a role for AI in becoming a personal tutor ‘transforming the role of the teacher’ was described and welcomed. Others felt AI would not pose a threat to the academic role:

*I think the academic world is more than knowledge; it’s about having a theory and the capacity to come up with a vision of the world, a theory or feelings about the world, is actually inherently human. And so I don’t think AI is going to steal the job of an academic. [Arts and Humanities]*

Participants imagined futures where AI plays a role in research, for example “astronomy, anything that requires the processing of vast amounts of data'' [Arts and Humanities 04]. Big data and AI were seen to be potentially transformative.

A third possibility was to build narratives about the everyday. Here mundane tasks and everyday decision making could become topics for stories.

*I think the way AI is working now can be useful for repetitive tasks, dull work, and dangerous work. [Social Science 01]*

Specific suggestions included, information retrieval, logistics, robots doing mundane tasks, such as watering grass and controlling heating systems. By focusing on mundane tasks, we could counter the narrative of fear around job losses. Instead, AI would support human practices. Indeed, there was a sense from over a quarter of the participants that AI will create jobs, not replace them.

*It will just create new jobs; people will want to maintain the robots. [Arts and Humanities 16]*

An alternative twist on this may come from the application of AI to particular domains. For instance, one participant suggested that AI will shift the role of teaching and in so doing create a new and exciting job:

*The hardest thing is to get the administrators and the teachers to come over to a completely different…they’ll have got a new job, it’s a different job. [Arts and Humanities 16]*

It follows that stories that detail and discuss the impacts on individuals and their adaptation to change could be a useful line. Rather than stoking fear, they could be accepting of change while championing human flexibility and growth.

Participants felt that stories might promote the role of AI in developing *relationships and community*: in bringing people together. As one such example, participants talked about a lack of stories which promote positive civic life:

*… possibilities for digital technologies or social technical processes to contribute positively and meaningfully to civic life… possibilities for new and emerging technologies to support social justice. [Social Science 19]*

AI was seen as something which could help cultural identities; *“help with place making, and processes that brought together institutions and communities, to redesign and transform public services.”* [Social Science 19] Participants described that while stories regularly refer to AI in relation to community there are often downsides:

*Often, you know, perhaps every day think wow, that’s amazing, that particular connection on the Twitter feed or the way that the hashtags work, that is creating possibilities of community at the very same time as its creating possibilities for trolling and for racism and for abuse of others. So I guess, you know, it’s not that it’s unwelcome in my house, it’s very welcome but it’s just that all the time those things are happening simultaneously. [Social Science 10]*

Some experts mentioned how AI could be used to strengthen relationships, build community, to ‘connect people’, ‘help us build better relationships’, using robots to support isolated individuals (e.g. care robots), and social networks. Another example that occurred frequently was dating.

Crucially, it was also explicit what AI should not do in this space: that it should not replace relationships between humans. This comes to the fore as one participant brings up the issue of mental health support:

*My kids are teenagers and they don’t even need their dad, never mind AI, they need me to talk to about their shit, and there’s no replacement, not even a human replacement. You know, very, very kind of particular human, yeah, activities that are very specific, so... I mean, I know there are sort of AI kind of experiments with mental health support and stuff but I just can’t see that working here. [Social Science 14]*

A connected issue is the relationship to the *environment*. For example, in agriculture and food stability, climate change and ecology:

*They’re doing a lot of good work in factories and increasingly in farms and in supply chain management and in postal processing and these are not places where either storytellers or lay people are busy very often so I mean there is a general lack of awareness about what happens in terms of how everything gets into our home and how civilisation functions and most of what AI does it does it in those spaces and so I think those are actually fairly I mean in some sense they are boring stories but in another sense it’s the way AI gets woven into how our civilisation is run these are just completely missing narratives. [Science 12]*

Many talked about uses of AI which could be seen to support the action against climate change. For example, one talked about how AI is helping to design plant-based products instead of animal-based products:

*I think meat, dairy, meat and dairy are such a huge problem on a global scale and I think the role AI plays in finding healthy t or convincing plant-based meat alternatives and dairy alternatives. [Social Science 01]aste*

Participants felt that to counter pessimistic and dystopian views of AI, health was an area where AI could be seen to help.

*I think healthcare probably is the area for me where you know, particularly combined with something like quantum, the benefits of it in terms of their visions, are huge. [Social Science 21]*

Almost all mentioned how AI can transform health particularly in the areas of care for the elderly, applications in psychology and infectious diseases. Other suggestions for new narratives related to the need to move the conversation away from the uncanny valley and robots in care, towards embracing possibilities to reduce patient loneliness, isolation, and pressure on public services. At the mundane level, stories could orient to the everyday wellbeing aspects of our lives, such as through the small things, like Fitbits and using timers to remind us to stand and stretch. Other examples of new narratives could include conversations about the role of human intelligence in the health space, such as using AI-enabled X-rays to find tumours.

*I mean the obvious answer is in the medical field, but that’s hard to say because actually I think there are very few tangible applications that are specifically aiming at the common good and that are not connected with economic purposes. [Science 01]*

Participants noted that of course, narratives that look to answer the biggest opportunities in health and social care, require thoughtful reflection to educate about the potential impacts of their implementation, and the underlying, but rarely publicly aired, business models.

Further, participants also talked about the possibilities for new and emerging technologies to support *social justice*, mitigating against societal biases through a process of bringing marginalised communities into the process of technology design (design justice) and empowerment.

*I think the question of design justice is a question for AI. What does it mean to proceed from a position of justice in that space? [Social Science 20]*

Importantly, participants felt that inclusivity and equality was paramount in telling stories about AI for justice:

*Who do these stories speak to, and what are the concerns that people live within their day-to-day lives? For it to be beneficial, people need to be put in the conditions to be able to benefit from it. And that requires that sort of social capabilities. [Science 02]*

The issue of algorithmic injustice was regularly referred to by all participants, focusing mainly around particular areas. All felt a deeper focus on narratives of social injustice could be used to build momentum in the movement toward a more just AI.

*That’s often what the narrative might be around object detection, facial recognition and so on, that you might well have a large percentage of people coming back and saying actually, yes, I would be willing to have that happen but in practice, what will happen is that those communities already at greatest risk of being stopped and searched by the police or being targeted as high risk will find that intensified through the use of the technology. [Social Science 10]*

In almost every instance, participants called for a need for stories which explained more about bias. As one participant put it, to *“put a magnifying glass on our own behaviour.”* [Social Science 11] Participants couched a way of weaving AI into how civilisation is run in terms of mobilising AI to reflect human virtues, cognisant that the technology is never neutral. These virtues could underpin stories of culture, social, justice, and freedom. For instance, one participant felt that from this position, the technology will mirror ‘good’ human virtues:

*If you talk to young people and you ask them what the future is going to be like, very few of them have stories of abundance, stories of leisure, stories of significant new freedom, significant new protections, new social contracts, all of these are very… I mean we have had periods in the past where these were highly imaginable and they made change happen, it wasn’t just them, right, I’m not blind to the way power operates but they are meaningful and I think we start there and the technology kind of will… technology will follow from them. [Arts and Humanities 14]*

A further area to explore might be AI and its relationship with spirituality. Participants described how there were missing AI narratives about the role AI can play with respect to *spiritual growth* and taking more time to tune into inner experience.

*It would be nice to be able to turn up the zen setting on my phone, without the phone even noticing that my heart rate is especially up. [Arts and Humanities 16]*

There was a sense that these notions were in conflict with the notion of personalisation which AI thrives on. In that it presents in ways, a false reality which could be seen to distract from the harsh realities of the world. In some senses, ‘tuning out’ noise from the world was seen as worth it, for a lot of participants. Notably, these responses might have been shaped by the context of the pandemic at the time of interviewing where there was increased focus on digital interaction.

*Those things from the core of our… you know, I mean it can be spiritual growth and it can be community growth and it can be creative growth, like all of those things you can imagine a world of much more significant abundance if you give people back time and if you give people back connections and if you give people back kind of you cut back on the amount of work they do. [Arts and Humanities 12]*

Finally, participants talked about the need for more nuanced approaches to narratives around economics and policy. Here, a more nuanced and truthful approach to storytelling was desired, acknowledging tensions between economics and wellbeing. Participants discussed how narratives ought to focus on nuanced explorations of growth, responsible innovation, austerity, Universal Basic Income, public services, the clash between AI and economic trajectories, economics vs wellbeing, the problems with solutionist narratives that pose that AI can solve intractable and impossible-to-frame political and social problems.

*I think what should be reflected is the clash between the development of AI and economic trajectories. [Science 01]*

Perhaps exacerbated by the pandemic, many participants reported the tension between economic measures such as GDP and more important and difficult-to-measure considerations such as people’s wellbeing. These tensions, they suggest, extend to the economy between developing responsible technologies and what we understand to be an economic imperative: “*it all has to change, because you can't have both”. [Social Science 20].*

From these accounts it was clear that these domains far extend the dichotomous views prevailing in the commonly told stories about AI. As one participant suggested, we need to move past the spectacular to focus on more pressing issues.

*We need more AI Futures to try and understand all of this mess because if your futures like superintelligence capture a lot of our attention but there are other ones and we need to be paying more attention to them. [Arts and Humanities 17]*

## Discussion

Widely retold dystopian stories portray AI as a risk to our livelihoods, safety, jobs and very existence. Whilst noting the inherent value of narratives of science fiction for their entertainment and speculative value, the issue for many relates to what happens when those narratives influence those in power and they are given credence beyond a role as entertainment, and when those with capital prefer to fund sensationalist stories and not stories about humans in an AI-enriched future. Explicit in our findings is the presence of established and well-known dominant narratives, often emphasising or embellishing one aspect of a story. For instance, the participants talk about a preoccupation with solutionism or AI for optimisation — but often with a view to optimizing ‘the wrong thing’. This trend continued with other narratives, including a tendency for dominant stories to focus on the negative connotations of robotics, for instance — e.g., job loss, or even fear of enfeeblement, at the expense of making more of their role in care and health. Interestingly, when the COVID-19 crisis hit, suddenly robots (who could not catch a virus) were welcomed more readily into our care environments, than prior to the pandemic. Other dominant narratives about superintelligence were largely dismissed by as an esoteric preoccupation of a privileged few closer to the realms of science fiction than the current state of technology.

This disconnect led many to describe concerns over ethics and responsibility in AI. Most concerns about dominant narratives were suggestive of a need to better interrogate and reflect on the design and development of AI. Indeed, despite momentum in the space of AI ethics (Jobin 2019), most participants held grave concerns about the motivations of big tech companies and ‘ethics washing’ (Applin and Flick 2021). This extends to notions of responsible storytelling. Participants urged that AI narratives ought to tell the tales of more realistic and accurate truths, relating to issues of social injustice and inequalities to shift perception and create positive change. This is in line with Coeckelbergh’s (2021) theorisation of responsible narratives.

Such is the shifting and contingent nature of AI narratives – vis a vis the ‘why’, the ‘who’ and the ‘how’ (Bory 2019). If narratives outlined are what we need in society, why don’t we have them? We argue that this is largely because of who is telling them and why. Our participants show that the communicative purpose of entertaining and educating and tendencies towards sensationalism run contrary to every day. As one of our participants claimed ‘these could be boring stories’ (p.16). Our research supports current work on AI narratives and suggests there are missing stories which could promote thinking about critical questions in all of the domains where AI is beneficial and where they seem to be harmful, to shed light on the many ethical and political dilemmas posed by AI – stories that speak to everyone, stories they can invest in. The reason we do not have them yet is perhaps that there is not the resource or recognition of a social justice motivation for collecting them, though we are getting closer to it, particularly in popular academic books in the mainstream. The inevitability of the hype associated with more general narratives, however is still tending toward polls or extremes. See, for instance a recent ‘glossy’ Guardian article on the British AI strategy, where ‘bright siding’ is a dominant feature of ‘getting the story right’ (Hare 2022).^3^

To do this, reflection on the relative nature and trends in storytelling; e.g., relative to groups of people, (Yee 2017), place (Dihal et al. 2021), time and panorama, as illustrated above, is vital. As Kim (2022) recently stated, the field must “broaden the purview of intelligent machines”, taking into account cultural and historical forces which shape public understanding of AI. To return to the concept of optimisation of ‘the wrong thing’, our participants stress the need to interrogate who decides what the wrong thing is – who is telling the story, what is the genre and the communicative purpose of the story. The latter may extend to associated imagery or sound in screen media where AI is portrayed (see Better Images of AI, 2021^4^; Chubb and Maloney 2021). In all cases, the creeping bias in both story and algorithms themselves is a high priority from the perspective of our participants (Noble 2018). This chimes with the increased momentum in AI ethics (Bryson 2020) and related issues of responsibility in narratives (Coeckelbergh 2021).

In moving toward new and niche narratives, participants were mindful of the need for collective effort (Berditchevskaia and Baeck 2020), emphasising the need for collective development of niche, novel, and nuanced stories and collective reflection and listening to stories (Dillon and Craig 2021). This would involve working with AI. These would sit ‘in-between’ contemporary dichotomies of utopia and dystopia. They would also identify areas (where narratives have power) in which AI does not have a role in human life, countering the blanket solutionist approach currently in vogue. Narratives can mobilise perception about where AI should *not* be applied as well as where it should be. In a recent report, UKRI (2020) mapped out a vision for transforming our world with AI. Outlining a number of domains for AI to seize it’s potential, our participants tended toward a similar view, but with a focus on wellbeing, community and society over economic growth (Bareis and Katzenbach, 2021) Despite the bright-sided narratives presented by our participants, they also felt AI presented a paradox with the potential for unintended negative consequences, e.g., in education and science (Chubb et al, 2021) and in providing solutions in society for instance with respect to risk management, immigration control and the justice system (Amoore, 2020).

In many senses, the missing narratives are missing for a reason. Principal narratives are being told by a narrow section of society, and therefore reflect the inherent social biases of that sector (Cave et al. 2019; O’Neil 2016). So too, the narratives we are commonly told about AI are heavily subjected to hype which can ‘obscure views of the future’ as Gemma Milne describes in her recent book (Milne, 2020). We see this, for instance, in the history of technology development: when a new technology is introduced it is met with sensationalization (Marvin 1990). The ‘ordinary’ bicycle or penny farthing for example was initially met with anxiety as it came to represent a particular form of aggressive and danger-seeking masculinity (Pinch and Bijker, 1984)^5^. Electricity inspired interest and concerns about ether and the spirits (Sconce, 2000) and most famously Mary Shelley’s Frankenstein promoted the idea of the intelligent killer robot. Such accounts position the new technology as a progressive narrative of changing attitudes of rejection and acceptance. Initially, and so currently, a new technology such as AI is mysterious and frightening and this opens up potential for extreme responses and views (Cave and Dihal, 2019). In relation to missing narratives, certain stories dominate to begin with, but as technology becomes accepted and integrated into everyday life these stories are likely to lose their hold and be replaced by diverse and more realistic alternatives.

What is prominent in our interviews is the need for stories which reflect community and promote social justice—an alternative form of the progressive seen in political responses to technologies. Here, it is noted that certain technologies marginalise certain groups and hence some technology stories and viewpoints are occluded and subordinated (Cave et al, 2020). One response is the reclaiming and appropriation of a technology by marginal groups (see for example Rosen’s 2002 discussion of the bicycle). Perhaps we need to focus more clearly on how those narratives are formed, and ‘from whom’ they emanate (Cave et al, 2020). Indeed, the extent to which these fictional narratives inform and engage with key social issues (Cave et al, 2019), such as ascribing a view of race and ‘whiteness’, is of ongoing concern and debate, not only through stories, but in wider attempts to decolonise AI.

In both cases we see missing narratives. Whereas in the first scenario we would anticipate a move towards acceptance and an emergence of the mundane and everyday understandings, in the second, this potentially never comes. Or at least it does not come without struggle. And so it is with AI. We could see the dominance of certain narratives as indicative of its early development—perhaps a necessary condition of almost any technological innovation. We might explain the twin extremes of dystopia and utopia as mere growing pains. Alternatively, we might understand the missing narratives as indicative of subordination and occlusion by powerful people. Perhaps there will never be stories about certain areas of AI use, precisely because they extend from marginalised positions of power (Miller, 2020).

One way to resolve the two alternative perspectives on missing narratives is to combine them. Maybe the progression of any technology acceptance is populated by political moments of critique and reflection. Perhaps, it is precisely those moments that help populate the everyday and spur calls for inclusion and extension into areas of relevance untraversed till that point. This is the space of missing narratives and their celebration and advocacy. It is through such progressive moves that we might move forward AI stories and storytelling.

## Conclusion

There are missing stories about AI. Stories highlighting the reality of this technology are urgently required, developed for and with a wide range of voices, across a range of domains. Some highlighted here chime with political efforts for AI futures, others go deeper. Particularly emphasized is that these narratives must align with human virtues or values. That is, that these stories ought to inspire and promote scenarios which extend beyond current power structures. These considerations might extend to addressing the role of science fiction in propagating the current dominant narratives and exploring whether we need different or diverse approaches in science fiction as well.

Stories can help with sense-making but they can also distort and distract. If the power of stories is mobilised for good, key actors will be encouraged to focus on the missing narratives as opposed to those which reinforce existing tropes unhelpful for public understanding and as inspiration for policy. Responsible narrative and storytelling that promotes public understanding of AI are particularly important. Our research suggests that a participatory approach to story-making, inclusive of individuals from varying backgrounds and ages can change the way we live alongside AI. Crucially, such an approach should not be blind to the way power operates and influences AI narratives (Hao 2021) and geopolitical aspects of AI ought to be considered, especially as new strategies on AI continue to emerge (HM Government, 2021), often without considering supporting narratives or awareness of the effect of dominant tropes. Going forward, there is a need to move toward a deeper reflection on the stories being told and in particular to consider the storyteller, genre and the communicate agenda or purpose when using story to inform public perception of AI. It is critical to consider who controls dominant narratives and who stands to gain from them. Efforts to make it harder (through scrutiny, regulation, and ethics) for those who benefit from the narratives to influence them ought to be a focus for the research and technology community at large.

To do this, further research focusing on these narrative features will be helpful, including broadening our participant sample to include a range of audiences. We note that the perspectives of our sample on the cultural significance of AI and "what is missing" have the potential to be skewed by their positions in the academy. The same interviews could be conducted with members of the general public, for instance and people from different cultural and geographical contexts. Future research could explore approaches by storytellers in literature and screen — focusing on fiction and documentary/non-fiction — in how they talk about AI. So too, audiences and public views could be further consulted and remain sensitive to changing notions of AI itself. Going forward there is a need to extend the question of narratives to consider not only who is creating them but who is featured in these stories and how we can be more inclusive in future storytelling. This might involve bringing in children and adults from a range of backgrounds to co-create accessible stories. Though out of scope of this paper, a final moment in AI narratives comes in the form of AI as storyteller. As Kate Crawford recently argued in her book Atlas of AI (2021) the shifting nature of what we mean by the term artificial intelligence and its capabilities is in flux and ‘that too is part of the story’.

Narratives that probe nuanced answers to these questions can help us get closer to broadly acceptable answers. Attention needs to be paid to the development of nuanced AI narratives that continue 'story-listening' beyond 'expert' voices toward collective, public participation, engage with the everyday and recognise the relative nature of narratives extending beyond the Anglophone West. Narratives are also relative to the domain of use they are describing.

There is a need to encourage and foster diverse storytellers, mindful of the way power operates. These narratives can take many forms, including forms that do not yet exist. Educating and empowering people to tell and deeply listen to compelling stories will promote the best possible futures for AI. To do that there has to be an opening and broadening of stories and storytellers that might shape public understanding. Further research must ask how we can begin to reshape the dynamics of public storytelling around AI to understand whether or not there really is an “AI story crisis”.

## Data Availability

Anonymised data can be made available upon request via ethics approval.

## Appendix 1

### Interview questions and structure (semi-structured guiding questions)

1. Background, discipline, level of interest, reason for involvement
2. What are the commonly told stories of AI? / dominant narratives from the perspective of the public, in your view?
3. To what extent if at all do these stories differ from reality? If so what differentiates them? where do they originate, who features in these stories? (probe gender/ politics/ tech/ marketing, science)
4. Are there any domains of use where you see an opportunity for AI?
5. How do you define /characterize AI? (categories and boundaries of AI)
6. Let’s take some time to imagine a future with AI
7. If ...you imagine a future in ‘the workplace’ or insert x - what role would AI play?
8. … Or imagine yourself at home - What role would AI be playing?
9. … Imagine the future of academic life… What role would AI be playing? … Imagine the future of games … What role would AI play?
10. …. Imagine the future of culture, interactive media and storytelling - what role would AI play?
11. Now how do you feel personally about the future you imagined?
  - Positive / negative?
12. So we have imagined a future with AI. How do we nurture the creativity required for AI to take the form you just described
  - How would you resource and create an infrastructure that would facilitate the technological developments required?
  - Within these structures and developments, who takes responsibility (needs to be involved) for ensuring a positive development of AI and its consequences?
  - How do we bring different perspectives together?
13. Finally, all things taken together, what can we learn going forward about AI - what stories can we tell about AI if any?
14. Any other comments

## Footnotes

1 UK National AI Strategy https://assets.publishing.service.gov.uk/government/uploads/system/uploads/attachment_data/file/1020402/National_AI_Strategy_-_PDF_version.pdf.

2 https://www.metaculus.com/questions/?show-welcome=true.

3 https://www.theguardian.com/commentisfree/2022/feb/13/the-rise-of-ai-could-be-a-great-british-story-but-lets-do-it-the-right-way.

4 Better Images of AI: https://blog.betterimagesofai.org/press-release-better-images-of-ai-launches-a-free-stock-image-library-of-more-realistic-images-of-artificial-intelligence/.

5 https://www.si.edu/stories/19th-century-bicycle-craze

## References

[CR1] Amoore L (2020). Cloud ethics.

[CR2] Applin SA, Flick C (2021). Facebook's Project Aria indicates problems for responsible innovation when broadly deploying AR and other pervasive technology in the Commons. J Responsible Technol.

[CR3] Bareis J, Katzenbach C (2021). Talking AI into being: The narratives and imaginaries of national AI strategies and their performative politics. Sci Technol Human Values.

[CR4] Benjamin R (2019). Race after technology: Abolitionist tools for the New Jim Code.

[CR5] Berditchevskaia & Baeck, 2020, The future of minds and machines . https://www.nesta.org.uk/report/future-minds-and-machines/ .Accessed 14 February 2022.

[CR6] Blom JN, Hansen KR (2015). Click bait: forward-reference as lure in online news headlines. J Pragmat.

[CR7] Bory P (2019). Deep new: the shifting narratives of artificial intelligence from Deep Blue to AlphaGo. Convergence.

[CR8] Braun V, Clarke V (2006). Using thematic analysis in psychology. Qual Res Psychol.

[CR9] Bryson JJ (2020). The artificial intelligence of the ethics of artificial intelligence. The Oxford handbook of ethics of AI. Computer Sci.

[CR10] Cave S, Dihal K (2019). Hopes and fears for intelligent machines in fiction and reality. Nature Machine Intelligence.

[CR11] Cave S, Dihal K (2020). The whiteness of AI. Philosophy Technol.

[CR12] Cave, S., Craig, C., Dihal, K., Dillon, S., Montgomery, J., Singler, B., & Taylor, L. (2018). *Portrayals and perceptions of AI and why they matter*. The Royal Society.

[CR13] Cave, S., Coughlan, K., & Dihal, K. (2019, January). " Scary Robots" Examining Public Responses to AI. In *Proceedings of the 2019 AAAI/ACM Conference on AI, Ethics, and Society* (pp. 331–337).

[CR14] Cave S, Dihal K, Dillon S (2020). AI narratives: A history of imaginative thinking about intelligent machines.

[CR15] Chubb, J., & Maloney, L. (2021) *AI what’s that sound*? Stories and Sonic framing of AI, https://blog.betterimagesofai.org/ai-whats-that-sound-stories-and-sonic-framing-of-ai/, Accessed 11 February 2022

[CR16] Chubb J, Cowling P, Reed D (2021). Speeding up to keep up: exploring the use of AI in the research process. AI & Soc.

[CR17] Coeckelbergh M (2021). Narrative responsibility and artificial intelligence. AI Soc.

[CR18] Crawford K (2021). The atlas of AI.

[CR19] Devlin K (2018). Turned on: Science, sex and robots.

[CR20] Dihal, K., Hollanek, T., Rizk, N., Weheba, N., & Cave, S. (2021). *Imagining a Future with Intelligent Machines A Middle Eastern and North African Perspective.* Canbridge: The Leverhulme Centre for the Future of Intelligence.

[CR21] Dillon S, Craig C (2021). Storylistening: Narrative evidence and public reasoning.

[CR22] Fast, E., & Horvitz, E. (2017, February). Long-term trends in the public perception of artificial intelligence. In *Proceedings of the AAAI Conference on Artificial Intelligence* (Vol. 31, No. 1).

[CR23] Felt U, Asveld L, Dam-Mieras R, Swierstra T, Lavrijssen S, Linse K, Hoven J (2017). Response-able practices” or new bureaucracies of virtue”: the challenges of making RRI work in academic environments. Responsible innovation.

[CR24] Hao, 2021 Stop talking about AI ethics. It’s time to talk about power. https://www.technologyreview.com/2021/04/23/1023549/kate-crawford-atlas-of-ai-review/ Accessed 29 June 2022

[CR25] Hare. S., (2022). The Rise of AI could be a great British Story retrieved from https://www.theguardian.com/commentisfree/2022/feb/13/the-rise-of-ai-could-be-a-great-british-story-but-lets-do-it-the-right-way , Accessed 15 February 2022

[CR26] Hermann I (2020). Beware of fictional AI narratives. Nature Machine Intelligence.

[CR27] HM Government, (2021). National AI Strategy https://assets.publishing.service.gov.uk/government/uploads/system/uploads/attachment_data/file/1020402/National_AI_Strategy_-_PDF_version.pdf, Accessed 27 September 2021

[CR28] Hudson AD, Finn E, Wylie R (2021). What can science fiction tell us about the future of artificial intelligence policy?. AI Soc.

[CR29] Jameson F (2005). Archaeologies of the future: The desire called utopia and other science fictions.

[CR30] Jasanoff S, Kim SH (2015). Dreamscapes of modernity.

[CR31] Jobin A, Ienca M, Vayena E (2019). The global landscape of AI ethics guidelines. Nat Mach Intell.

[CR32] Kang M (2011). Sublime dreams of living machines.

[CR33] Katz Y (2020). Artificial Whiteness: Politics and Ideology in Artificial Intelligence.

[CR34] Kearney R (2002). On Stories.

[CR35] Kelley, P. G., Yang, Y., Heldreth, C., Moessner, C., Sedley, A., Kramm, A., & Woodruff, A. (2021, July). Exciting, Useful, Worrying, Futuristic: Public Perception of Artificial Intelligence in 8 Countries. In *Proceedings of the 2021 AAAI/ACM Conference on AI, Ethics, and Society* (pp. 627–637).

[CR36] Kim MS (2022). Meta-narratives on machinic *otherness*: beyond anthropocentrism and exoticism. AI & Soc.

[CR37] Kress N (2001). Computer virus. *Asimov’s Science*. Fiction.

[CR38] LaGrandeur K (2013). Androids and intelligent networks in early modern literature and culture: artificial slaves.

[CR39] Marvin C (1990). When old technologies were new: Thinking about electric communication in the late nineteenth century.

[CR40] Mayor A (2018). Gods and robots: myths, machines, and ancient dreams of technology.

[CR41] McAdams DP, Guo J (2015). Narrating the generative life. Psychol Sci.

[CR42] Mead GH, Dewey J (1959). The philosophy of the present. Murphy AE.

[CR43] Miles MB, Huberman AM, Saldana J (2014). Qualitative data analysis: a methods sourcebook.

[CR44] Miller K, Jackson M, Shelly M (2020). A Matter of Perspective: Discrimination, Bias, and Inequality in AI. Legal Regulations, Implications, and Issues Surrounding Digital Data.

[CR45] Milne G (2020). Smoke & Mirrors: how hype obscures the future and how to see past it.

[CR46] Noble SU (2018). Algorithms of oppression: how search engines reinforce racism.

[CR47] O'Neil C (2016). Weapons of math destruction: How big data increases inequality and threatens democracy.

[CR48] Ouchchy L, Coin A, Dubljević V (2020). AI in the headlines: the portrayal of the ethical issues of artificial intelligence in the media. AI & Soc.

[CR49] Pinch TJ, Bijker WE (1984). The social construction of facts and artefacts: or how the sociology of science and the sociology of technology might benefit each other. Soc Stud Sci.

[CR50] Robbins (2016) Artificial Intelligence: Gods, egos and Ex Machina, https://www.theguardian.com/science/the-lay-scientist/2016/jan/26/artificial-intelligence-gods-egos-and-ex-machina, Accessed 21 September 2021

[CR51] Rosen P (2002). Up the vélorution: appropriating the bicycle and the politics of technology. Appropriating Technology: Vernacular Science and Social Power.

[CR52] Samuel G, Diedericks H, Derrick G (2021). Population health AI researchers’ perceptions of the public portrayal of AI: a pilot study. Public Underst Sci.

[CR53] Sconce J (2000). Haunted media: Electronic presence from telegraphy to television.

[CR54] Slota SC, Fleischmann KR, Greenberg S, Verma N, Cummings B, Li L, Shenefiel C (2020). Good systems, bad data? Interpretations of AI hype and failures. Proceedings of the Association for Information Science and Technol.

[CR55] Stathoulopoulos, K., & Mateos-Garcia, J. C. (2019). Gender diversity in AI research. *Available at SSRN 3428240*.

[CR56] Stratton & Milford (2017) The future of artificial intelligence: two experts disagree. *The Conversation*, https://theconversation.com/the-future-of-artificial-intelligence-two-experts-disagree-79904, Accessed 10 April 2021

[CR57] Truitt ER (2015). Medieval robots.

[CR58] UKRI (2020) Transforming our world with AI. https://www.google.com/url?q=https://www.ukri.org/about-us/what-we-do/strategies-and-reviews/ai-review-transforming-our-world-with-ai/&sa=D&source=editors&ust=1632743965461000&usg=AOvVaw0voNVkr4NIrgpff3LW_YO-

[CR59] Vallor S (2016). Technology and the virtues: A philosophical guide to a future worth wanting.

[CR60] Vallor, S. (2021) Challenging and redrawing framings of technology to serve human flourishing and justice. https://edinburgh-innovations.ed.ac.uk/2021/08/04/mobilising-the-intellectual-resources-of-the-arts-and-humanities/, Accessed 28 Sept 2021

[CR61] Wade M (2018). Virtuous play: the ethics, pleasures, and burdens of brain training. Science as Culture.

[CR62] Ward M (2018). Seeming Human: Artificial Intelligence and Victorian Realist Character.

[CR63] Yee S (2017). You bet she can fuck”–Trends in Female AI Narratives within Mainstream Cinema: Ex Machina and Her. Ekphrasis Images, Cinema, Theory Media..

[CR64] Zhang, B., & Dafoe, A. (2020, February). US public opinion on the governance of artificial intelligence. In *Proceedings of the AAAI/ACM ConferSence on AI, Ethics, and Society* (pp. 187–193).

